# Necrotizing Enterocolitis in Term Neonates: A Retrospective Chart Review from Children’s Medical Center, Tehran (2020–2023)

**DOI:** 10.34172/aim.34874

**Published:** 2025-10-01

**Authors:** Kayvan Mirnia, Maryam Saeedi, Amir Ali Ahrabi, Razieh Sangsari, Sepideh Poshtdar, Abdul Latif Panhwer

**Affiliations:** ^1^Children’s Medical Center, Pediatric Center of Excellence, Faculty of Medicine, Tehran University of Medical Sciences, Tehran, Iran; ^2^Faculty of Medicine, Tehran University of Medical Sciences, Tehran, Iran; ^3^Eye Research Center, Farabi Eye Hospital, Tehran University of Medical Sciences, Tehran, Iran

**Keywords:** Gastrointestinal diseases, Infant, Necrotizing enterocolitis

## Abstract

**Background:**

Necrotizing enterocolitis (NEC) is one of the most life-threatening conditions affecting neonates admitted to the neonatal intensive care unit (NICU). Prematurity and low birth weight are the most widely recognized risk factors. However, NEC can also occur in term neonates. This study investigated the incidence, clinical presentation, and underlying medical conditions associated with NEC in term neonates at a tertiary referral children’s hospital in Tehran, Iran.

**Methods:**

A retrospective review was conducted on term neonates diagnosed with NEC from 2020 to 2023. Data were collected on demographics, clinical presentation, laboratory findings, underlying medical conditions, treatment, and outcomes.

**Results:**

Of 975 term neonates admitted to the NICU during the study period, 33 were diagnosed with NEC, yielding an incidence of 3.38% (95% CI: 2.33–4.72). The mean birth weight was 3.15±0.57 kg, and NEC symptoms appeared at a mean age of 18.3±8.1 days. Abdominal distention was the most common initial symptom (78.8%). A history of herbal medication intake was reported significantly higher compared to non-NEC term neonates (*P*=0.02). Medical management was sufficient in 87.9% of cases, and no mortality was observed. The median hospital stay was 9 days (IQR: 7–13).

**Conclusion:**

This study highlights a notable incidence of NEC among term neonates in a tertiary Iranian NICU and identifies herbal medication intake as a potential risk factor. These findings underscore the importance of early recognition and culturally sensitive preventive strategies in term infants, especially in regions where herbal remedies are commonly used.

## Introduction

 Necrotizing enterocolitis (NEC) is a devastating condition and a leading cause of morbidity and mortality among neonates in the neonatal intensive care unit (NICU). It is characterized by inflammation and necrosis of the intestinal tract, which can progress to perforation and death.^1,2^ While NEC is predominantly a disease of prematurity, affecting approximately 7% of very low birth weight infants globally, its occurrence in term neonates is a rare but critically important clinical entity.^1,3^ Although NEC primarily affects premature infants, only a small fraction—estimated between 0.5% and 1.5%—of all NEC cases occur in full-term newborns. Because of this rarity, the clinical characteristics and risk factors in term infants are not well defined. Term neonates who develop NEC often have distinct predisposing conditions that differentiate them from their premature counterparts, leading to ongoing debate about the pathophysiology of the disease in this group. While well-established risk factors for premature NEC include low birth weight and immature gut function, the etiology in term infants is more strongly linked to specific underlying medical conditions. These include congenital heart disease, perinatal asphyxia, congenital malformations, and certain feeding practices.^3-6^ Despite the potential for a different clinical picture and disease course, data on the presentation, severity, and overall outcomes of NEC in term patients are infrequently documented, particularly within specific regional populations like Iran. The scarcity of such data represents a critical knowledge gap. Given this context, the primary objective of this retrospective study was to address this gap by characterizing the clinical presentation, identifying associated medical conditions and risk factors, and describing the outcomes of NEC in term neonates at a tertiary referral children’s hospital in Iran. Our findings aim to provide a clearer understanding of this rare and life-threatening condition in this unique patient population.

## Materials and Methods

###  Study Design and Setting

 This study was a retrospective chart review conducted at the NICU of the Children’s Medical Center, a tertiary referral hospital affiliated with Tehran University of Medical Sciences, Tehran, Iran. The review covered medical records from March 2020 to August 2023.

###  Study Population

 Eligible participants included term neonates (gestational age ≥ 37 weeks) admitted to the NICU within the first 28 days of life who were diagnosed with NEC by a board-certified neonatologist or pediatric surgeon. NEC diagnosis was established using the Modified Bell’s staging criteria, which incorporate systemic, intestinal, and radiologic findings.

- ***Stage I (Suspected NEC):*** Characterized by nonspecific systemic signs such as temperature instability, lethargy, apnea, and bradycardia, along with mild gastrointestinal symptoms including feeding intolerance, mild abdominal distension, and emesis. Radiologic findings are typically normal or show mild ileus.
- ***Stage II (Definite NEC):*** Includes more pronounced gastrointestinal signs such as absent bowel sounds, abdominal tenderness, and possible presence of occult or gross blood in stool. Radiologic evidence may show pneumatosis intestinalis or portal venous gas. Laboratory abnormalities may include thrombocytopenia and metabolic acidosis.
- ***Stage III (Advanced NEC):*** Defined by signs of systemic deterioration including hypotension, acidosis, disseminated intravascular coagulation (DIC), and respiratory failure. Gastrointestinal findings may include marked abdominal distension, peritonitis, and evidence of intestinal perforation. Radiologic imaging may reveal pneumoperitoneum.^5^

Staging was performed by a board-certified neonatologist or pediatric surgeon based on the constellation of clinical, laboratory, and imaging findings. This classification guided both medical and surgical management decisions throughout the study period. Neonates with other gastrointestinal disorders or lacking parental informed consent were excluded from the study.

###  Data Collection

 Clinical data were obtained from multiple institutional sources at the Children’s Medical Center in Tehran, Iran. These included: NICU admission and discharge registries, surgical consultation and operative reports, laboratory information system for biochemical and hematologic parameters, radiology reports for imaging findings relevant to NEC staging, nursing and physician documentations for feeding practices and symptom onset. A standardized data collection form was developed by the research team to ensure consistency and completeness. Trained neonatology fellows and pediatric residents, under supervision of the principal investigator, manually reviewed and extracted relevant data from the above sources. Each record was cross-verified for accuracy and completeness. Data were anonymized and de-identified prior to analysis to maintain patient confidentiality. Variables included demographic characteristics, clinical presentation of NEC, laboratory parameters, underlying medical conditions, history of medication use (including herbal remedies), treatment modalities, length of hospital stay, and clinical outcomes. Laboratory markers of acute illness included C-reactive protein (CRP), white blood cell (WBC) count and differential, hemoglobin, platelet count, blood urea nitrogen (BUN), serum creatinine, electrolytes, arterial blood pH, and base excess. Rectal full-thickness biopsies were performed in cases with suspected Hirschsprung’s disease.

###  Management Protocol

 All neonates received standard supportive care, including intravenous fluid resuscitation, broad-spectrum antibiotics, cessation of enteral feeding, and nasogastric decompression. Additional interventions (such as blood product transfusion, parenteral nutrition, cardiorespiratory support, or surgical treatment) were administered based on clinical indications. Surgical intervention was considered in cases of confirmed intestinal perforation, peritonitis, obstruction, Hirschsprung’s disease or clinical deterioration unresponsive to medical therapy.

###  Statistics

 Descriptive statistics were utilized to report the quantitative variables using SPSS version 27, including mean, median, standard deviation, and interquartile range (IQR) according to normality tests. Qualitative data were presented as numbers and percentages. Fisher’s exact test was performed to analyze the association between categorical variables. In this study, a *P* value less than 0.05 was considered statistically significant.

## Results

**Figure 1:**
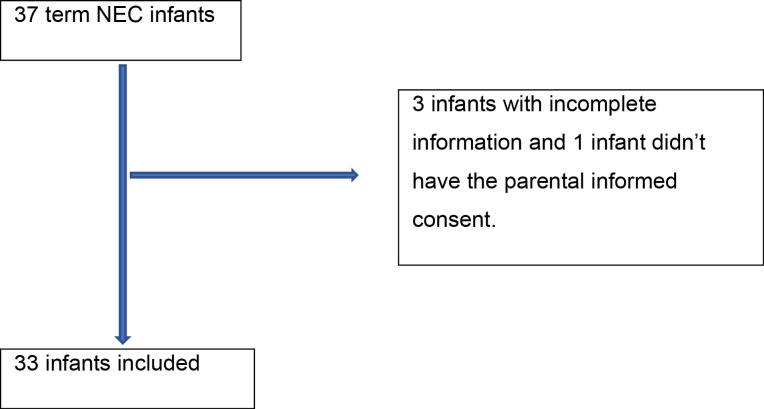
Flow Diagram of Infant Selection

 Between March 2020 and August 2023, a total of 975 term patients were admitted to the NICU of the Children’s Medical Center. Among these patients, 37 were diagnosed with NEC, of whom four infants were excluded from the study due to incomplete information or not having the parental informed consent, resulting in an overall incidence of 3.38% over the three-year period (Figure 1). Of the diagnosed patients, seven were classified as Bell stage 2. None of the patients were classified as stage 3. The demographic characteristics and clinical presentations of NEC in these patients are summarized in Table 1. The most common initial presentation of NEC was abdominal distention, observed in 78.8% of patients, followed by jaundice (42.4%), bilious vomiting (42.4%), poor feeding (33.3%), and diarrhea (12.1%).

**Table 1:** Patients’ Demographic Data and Initial Symptoms at of NEC Onset Among Patients

**Demographic characteristics**	**Value**
Sex (male) (%)	19 (57.5%)
Gestational age at birth (wk) (mean ± SD)	38.0 ± 0.7
Birth weight (kg) (mean ± SD)	3.15 ± 0.57
Age of NEC onset (days) (mean ± SD)	18.3 ± 8.1
Admission weight (kg) (mean + SD)	3.34 ± 0.62
C-section delivery route (%)	20 (61%)
**Clinical presentation of NEC (%)**	**Value**
Abdominal distention	26 (78.8%)
Jaundice	14 (42.4%)
Bilious vomiting	14 (42.4%)
Poor feeding	11 (33.3%)
Diarrhea	4 (12.1%)

Table 2 presents the initial laboratory data of the patients. All lab results were within normal ranges, except for arterial pH, which indicated acidosis (pH < 7.35) in ten patients (30%). Elevated levels of CRP ( > 6 mg/L) were observed in only five cases (15%). Two patients exhibited leukopenia (WBC < 5 × 10^9^/L), while none showed leukocytosis (WBC > 25 × 10^9^/L). As shown in Table 3, the most common underlying medical condition was the intake of herbal medications (45%), followed by unknown factors (21%) and cow’s milk intake (15%). Ten patients had multiple underlying conditions.

**Table 2:** Results of Hematology, Electrolytes, and Acid-base Lab Tests Obtained from NEC Patients

**Lab data of patients with NEC**	**Value**
WBC	
Mean ± SD	10.1 ± 3.9 10^9^/L
Median, IQR	9.6, 8.0-11.7 10^9^/L
Differential neutrophil count (mean ± SD)	31.5 ± 16.5 %
Differential lymphocyte count (mean ± SD)	54.9 ± 16.7 %
Hemoglobin (mean ± SD)	13.6 ± 2.5 g/dL
PLT (mean ± SD)	359 ± 158 10^9^/L
CRP (median, IQR)	1, 1-3 mg/L
BUN (median, IQR)	6.4, 4.8-11.0 mg/dL
Cr (median, IQR)	0.47, 0.38-0.60 mg/dL
pH (median, IQR)	7.39, 7.0-7.4
Base excess (median, IQR)	-3, (-4.3) -(-0.2)
Na (mean ± SD)	136 ± 5 mmol/L
K (mean ± SD)	4.8 ± 0.6 mmol/L

**Table 3:** Underlying Medical Conditions Among 33 NEC Patients.

**Underlying medical conditions**	**No. (%)**	***P*** ** value**
History of herbal medication intake	15 (45%)	> 0.05
Mangosteen	6 (18%)	
Cotoneaster	3 (9%)	
Honey	1 (3%)	
Mint extract	1 (3%)	
Unknown herbal medication/oil	4 (12%)	
Cow’s milk Allergy	5 (15%)	
Hirschsprung's disease	4 (12%)	
Other condition	5 (15%)	
Anemia	1 (3%)	
Viral infection	1 (3%)	
Asphyxia	1 (3%)	
Severe dehydration	1 (3%)	
Immature ganglion	1 (3%)	
Unknown	7 (21%)	

 Among the 33 term neonates with NEC, 15 cases (45%) had a history of herbal medication use. We performed subgroup analyses to evaluate potential associations between herbal intake and clinical outcomes. Fisher’s exact tests showed no statistically significant association between herbal medication use and disease stage (Bell I vs. II, p = 0.65), age of onset ( ≤ 7 days vs. > 7 days, *P* = 1.00), or the need for surgical intervention (*P* = 0.29). No mortality occurred in either group. Also, among the 33 term neonates with NEC, 10 were suspected to have Hirschsprung’s disease, with four of these confirmed by rectal biopsy. A total of 29 cases required only medical treatment, while surgery was indicated in four patients with Hirschsprung’s disease (12%). No mortality was observed, and all 33 cases had a median hospital stay of 9 days (IQR: 7–13 days) and completed their recovery.

## Discussion

 NEC is the most prevalent life-threatening cause of gastrointestinal-related mortality and morbidity in the NICU. Only 10% of all NEC cases occur in term neonates, and as a result, this group remains less studied.^6,7^ To the best of our knowledge, this is the largest study on term patients diagnosed with NEC upon NICU admission, detailing their incidence, clinical presentation, and underlying medical conditions. The incidence of NEC among term patients in the NICU was found to be 3.38% in this study, highlighting the need for pediatricians to be vigilant and ensure prompt diagnosis in this population. NEC has traditionally been associated primarily with preterm or low-birth-weight neonates.^8^ Previous reports indicate that the overall incidence rates of NEC in the NICU (including both preterm and term neonates) range from 1% to 5%. However, in term patients specifically, the reported incidence is much lower, ranging from 0.016% to 0.071%, although there has been an increasing trend in recent years.^9^ The high incidence observed in the current study may be partially attributed to our center being a tertiary referral children’s hospital in Iran. Additionally, a contributing factor could be the tendency among families to administer herbal medicines to newborns. ln this study, 45% of neonate with NEC had a history of herbal medication use. Although within-group differences were not statistically significant, the high prevalence of herbal medication intake among NEC patients is a noteworthy finding. These herbal remedies are often prescribed by traditional medicine practitioners and used by families, particularly in cases of jaundice, which is a common underlying condition. In this study, the most frequent presentation of NEC was abdominal distension. Additionally, jaundice was observed in 42.4% of our patients during clinical examinations. A previous study indicates that hyperbilirubinemia reduces the risk of NEC due to its antioxidant activity.^10^ Notably, nearly 60% of our cases with jaundice had a history of taking herbal medications, primarily mangosteen and cotoneaster. We speculate that these patients may have been administered the herbal medications after the initial presentation of neonatal jaundice, which could have contributed to the subsequent development of NEC. Among the frequently prescribed herbal medications discussed in this study, *Garcinia mangostana* L., commonly known as mangosteen, is a tropical evergreen tree distinguished by its smooth, leathery leaves.^11^ In tropical regions, the pericarp of the mangosteen has traditionally been used to treat conditions such as abdominal pain, dysentery, diarrhea, infected wounds, and chronic ulcers. Mangosteen is rich in biologically active compounds, including xanthones, tannins, and various vitamins. Numerous studies have demonstrated that mangosteen possesses anti-inflammatory, antioxidant, antihistamine, and various other pharmacological properties.^12^ Notably, anti-angiogenesis is also part of its repertoire. The study by Wihastuti et al found that mangosteen significantly reduced vasa vasorum angiogenesis by inhibiting the pathways associated with H_2_O_2_, HIF-1α, NF-κB, and Inos.^13^ The subsequent decrease in NO production can ultimately lead to hypoxia and ischemia,^14^ which is the hypothesized mechanism for the occurrence of NEC in term neonates. Additionally, the history of intake of other herbal medications, such as cotoneaster, honey, and mint extracts, should be noted and warrants further evaluation. Another possible etiology that has rarely been discussed is Hirschsprung’s disease. Raboei first demonstrated the association between Hirschsprung’s disease and NEC in term patients. She conducted a retrospective study on NEC patients who underwent surgical treatment and found that 4 out of 17 term patients had aganglionosis. This suggests a high prevalence of underlying Hirschsprung’s disease (12%) as a potential predisposing factor for the occurrence of NEC among term neonates.^15^ One possible explanation for this association may be the excessive growth of *Proteobacteria*, which has been observed in both NEC and Hirschsprung’s disease.^16^ Given this information, pediatricians should remain vigilant for Hirschsprung’s disease when assessing term infants with NEC and perform relevant physical examinations. Five of our patients (15%) had an intake of cow’s milk, which is the most prevalent cause of allergy in infants during their first year of life. This often results from infant formula or breast milk from mothers who consume cow’s milk or dairy products.^17^ In a randomized controlled trial involving 207 infants, Sullivan et al concluded that exclusive human milk feeding is associated with a reduced incidence of NEC compared to diets that include bovine milk products.^18^ Many other studies have also reported an association between the intake of cow’s milk and the development of NEC.^19-21^ Reported literature indicates that the average onset of NEC among term patients ranges from 5 to 9 days after birth.^6,22^ However, we could not confirm previous findings that early-onset NEC is predominant in term patients, as the mean age of onset in our study was 18.3 ± 8.1 days. This discrepancy may be partly due to our center being a tertiary referral children’s hospital, and we suspect that the potential use of herbal medications for prolonged jaundice could also contribute to this later onset. Additionally, a few other studies have reported late-onset NEC in term patients. For example, in the study by Hadian et al in Iran on term infants with congenital heart disease, the mean age of NEC onset was 22.39 ± 44.48 days.^4^ In another study, Short et al^23^ examined 39 term patients, of whom 11 (28.2%) presented with late-onset NEC, defined as occurring after 7 days of age, while the remaining cases were classified as early-onset NEC, occurring within the first 7 days. They concluded that late-onset NEC in full-term patients is associated with higher mortality rates compared to early-onset cases. In contrast, our current study identified seven early-onset cases and 26 late-onset cases (21% vs.79%), with no reported mortality in either group. The only statistically significant difference observed between early-onset and late-onset cases in our study was that hemoglobin levels were lower in the late-onset group (15.3 g/dL compared to 13.1 g/dL; *P* = 0.05). This difference may be attributed to the age of the patients in each group. Furthermore, other variables and outcomes did not show significant differences between the two groups. Several studies have identified that NEC has a multifactorial etiology. One proposed factor is the transmission of bacteria to the neonate’s intestines during delivery. Li et al found that vaginal delivery is associated with a higher incidence of early-onset NEC in term patients compared to late-onset NEC (45.3% vs. 34%, *P* = 0.05).^24^ Additionally, another study among preterm infants concluded that vaginal delivery is an independent risk factor associated with early-onset NEC.^25^ However, we were unable to confirm these results in the current study, as 61% of the neonates were born via C-Section delivery with no significant difference observed between late-onset and early-onset NEC based on the mode of delivery (*P* = 0.2). In contrast, Sdona et al found that cesarean delivery was significantly more common in full-term and late-preterm neonates with NEC.^26^ A possible mechanism is that epidural anesthesia may lead to a temporary reduction in blood pressure, which could impair placental blood flow and fetal circulation.^9^ Additionally, a robust study conducted by Son et al demonstrated that the mode of delivery did not significantly influence the occurrence of NEC occurrence in preterm neonates.^27^ Further research is needed to clarify the impact of delivery mode on the various onset ages of NEC, thereby improving prevention and treatment strategies for this condition in term neonates. There has been ongoing debate regarding the differences in the pathophysiology of NEC between term and preterm neonates. In preterm neonates, the development of NEC is often attributed to an inflammatory process resulting from intestinal immaturity and a distinct microbiome. In contrast, in term neonates, the underlying medical conditions that are commonly identified during clinical assessment for NEC diagnosis are typically indicative of a hypoxic and ischemic state that can trigger the condition. These conditions include asphyxia, cyanotic congenital heart disease, sepsis, meconium aspiration syndrome, and the need for respiratory support.^28-30^ Abbo et al found that maternal or intrinsic disease was present in 81.5% of full term neonates with NEC.^4^ In the study conducted by Martinez-Tallo et al, only 12.5% of term neonates with NEC had no identifiable risk factors.^31^ Additionally, up to 66% of term neonates with NEC had co-existing congenital diseases in another study.^32^ Our study revealed that 88% of these patients had an underlying medical condition, with the primary factors being herbal medication intake and cow’s milk allergy. Laboratory examinations showed no significant abnormal findings, except for acidosis (pH < 7.35) in 30.3% of the cases and elevated CRP levels ( > 6 mg/L) in only 15.1% of the neonates. Metabolic acidosis has been reported to be associated with poor prognosis in patients with NEC patients, leading to higher mortality rates.^33^ Additionally, other studies have indicated that laboratory data in term patients with NEC often do not present characteristic findings. In many cases, low CRP and normal WBC counts suggest that an inflammatory process may not be the primary mechanism behind the occurrence of NEC in term neonates. Our study had limitations similar to those reported in previous studies, including sample size, study duration, inherent limitations of retrospective studies, and lack of detailed information regarding feeding practices, specifically breast versus formula feeding.

 This is a retrospective design, which includes the potential for incomplete data on patient feeding practices. Additionally, as a single-center study in a tertiary referral hospital, our findings may not be generalizable to the broader population. The high incidence rate observed likely reflects a referral bias, and the descriptive statistics used in the study do not allow us to establish causal relationships.

## Conclusion

 This study provides valuable, descriptive data on NEC in term neonates from a tertiary referral center in Iran, a region with limited published data on this topic. We highlight a high incidence of NEC in this population and identify potential a later age of onset and the high prevalence of herbal medication intake among NEC patients is a noteworthy finding. From a clinical perspective, our findings underscore the critical need for pediatricians to be vigilant for NEC in term infants, particularly those presenting with jaundice and a history of herbal medication use.
